# Supplementary material for: Predicting adherence to postdischarge malaria chemoprevention in Malawian pre-school children: A prognostic multivariable analysis
Source: PLOS Glob Public Health. 2023 Apr 17;3(4):e0001779. doi: 10.1371/journal.pgph.0001779 (PMC10109490; doi:10.1371/journal.pgph.0001779)
Supplement: S1 Text — (DOCX) [file pgph.0001779.s001.docx]

**Kühl et al: Predicting adherence to**

**postdischarge antimalarials in Malawian pre-school children**

**Supplementary Material, S1 Text**

**S1 Text:** Summary of methods and results behind the index-variable for households’
socio-economic status (SES)

Using the Stata 17 software package, we developed a socio-economic index for the 357 households from the PDMC trial in Malawi that were included in this predictor analysis [1]. Initially, 88 variables (incl. sub-categories) that included a range of household features and characteristics that potentially reflect a household’s economic status were considered (Table S1). Items accessible to or owned by less than 5% or more than 95% of households were excluded in order to strengthen the comparability between relatively wealthy and relatively poor households, leaving 27 variables included. An initial principal component analysis (PCA) was conducted where all households were awarded a relative score summarizing their households’ assets. Ordered in ascending order and separated in quintiles, the resulting index showed a skewed shape with the first four quintiles at relatively similar levels and only indicating for the fifth quintile a substantially higher relative socio-economic status. We adjusted the analysis to iteratively reduce multicollinearity: we created a correlation matrix and tested excluding variables with near perfect and very low correlation (>0.9 and <0.1 multiple correlations). The resulting index of 11 variables showed more heterogeneity while remaining skewed This index-value was ranged and divided in quintiles, too, and included in the predictor analysis. (Figures S1, S2).
