# Supplementary material for: Predicting adherence to postdischarge malaria chemoprevention in Malawian pre-school children: A prognostic multivariable analysis
Source: PLOS Glob Public Health. 2023 Apr 17;3(4):e0001779. doi: 10.1371/journal.pgph.0001779 (PMC10109490; doi:10.1371/journal.pgph.0001779)
Supplement: S1 Table — (DOCX) [file pgph.0001779.s002.docx]

**Kühl et al: Predicting adherence to**

**postdischarge antimalarials in Malawian pre-school children**

**Supplementary Material, S1 Table**

**S1 Table:** Overview of variables considered in the Predictor analysis

| **Variables** | **Variables included after excluding items available/used by <5% or >95% of households**  **(X: included)** | **Correlation adjustment**  **(X: included)** |
| --- | --- | --- |
| **The household or one household member has (yes/no):** | | |
| - a clock/watch | X | X |
| - a radio | X | X |
| - a black and white TV |  |  |
| - a colour TV |  |  |
| - a mobile Phone | X | X |
| - a non-mobile Phone |  |  |
| - a refrigerator |  |  |
| - a freezer |  |  |
| - a generator/inverter |  |  |
| - a solar panel | X | X |
| - a washing machine |  |  |
| - a computer |  |  |
| - a tractor |  |  |
| - a digital camera |  |  |
| - a non-digital camera |  |  |
| - a video deck |  |  |
| - a VCR/DVD |  |  |
| - a sewing machine |  |  |
| - a bed | X | X |
| - a table | X | X |
| - a cabinet/cupboard |  |  |
| - a fan |  |  |
| - a cassette player |  |  |
| - a plow |  |  |
| - a grain grinder |  |  |
| - a hammer mill |  |  |
| - candles | X |  |
| - kerosene | X |  |
| - a bicycle | X |  |
| - a motorcycle/scooter |  |  |
| - an animal drawn cart |  |  |
| - a car or truck |  |  |
| - a boat with motor |  |  |
| - a boat |  |  |
| Variables regarding the home build and resources: | | |
| - number of rooms |  |  |
| - number of household members per sleeping room | X |  |
| - type of toilet used by household   1 "Flush toilet"  2 "Pit latrine"  3 "Dug-out pit with roof"  4 "Dug-out pit without roof" 5 "None"  6 "Does not wish to disclose" 7 "No facility, bush, outdoor" | 2 "Pit latrine"  3 "Dug-out pit with roof"  4 "Dug-out pit without roof" |  |
| - toilet is shared with other households (yes/no) | X |  |
| - the type of fuel mainly used for cooking is   1"Electricity"  2"LPG/ natural gas"  3"Biogas"  4"Kerosene"  5"Coal, lignite"  6"Charcoal"  7"Wood/firewood"  8"Straws/Shrubs/grass"  9"Agricultural crop residue"  10"Animal Dung"  11"No food cooked in household" | 3"Biogas"  4"Kerosene" | 4"Kerosene" |
| - the main material of your roof is   1"Grass"  2"Iron sheets"  3"Clay"  4"Tiles"  5"Concrete"  6"Plastic Sheeting"  7"Does not wish to disclose"  8"Does not know" | 1"Grass"  2"Iron sheets" | 1"Grass" |
| - the main material of your main walls is   1"Grass"  2"Mud (Yomata)"  3"Compacted Earth (Yamdindo)"  4"Mud bricks (unfired)"  5"Burnt bricks"  6"Concrete"  7"Wood"  8"Iron sheets"  9"Does not wish to disclose"  10"Does not know" | 2"Mud (Yomata)"  3"Compacted Earth (Yamdindo)"  4"Mud bricks (unfired)" | 4"Mud bricks (unfired)" |
| - the main floor material in your house is   1"Earth/sand"  2"Smoother Mud"  3"Smooth cement"  4"Wood"  5"Tile"  6"Does not wish to disclose"  7"Does not know" | 1"Earth/sand"  2"Smoother Mud"  3"Smooth cement" | 2"Smoother Mud"  3"Smooth cement" |
| Variables regarding agricultural land | | |
| - the household owns agricultural land (yes/no) |  |  |
| - the size of the land in acres (alt. football fields) |  |  |
| **Variables regarding livestock** |  |  |
| - the household owns any livestock (yes/no) | X |  |
| - number of "milk cow/bull |  |  |
| - number of "sheep" |  |  |
| - number of "horse/donkey/mule" |  |  |
| - number of "chicken" | X |  |
| - number of "goats" | X |  |
| - number of "pigs" |  |  |
