# Supplementary material for: Predicting adherence to postdischarge malaria chemoprevention in Malawian pre-school children: A prognostic multivariable analysis
Source: PLOS Glob Public Health. 2023 Apr 17;3(4):e0001779. doi: 10.1371/journal.pgph.0001779 (PMC10109490; doi:10.1371/journal.pgph.0001779)
Supplement: S1 Fig — (DOCX) [file pgph.0001779.s003.docx]

**Kühl et al: Predicting adherence to**

**postdischarge antimalarials in Malawian pre-school children**

**Supplementary Material, S1 Fig**

**S1 Fig:** The eigenvalues for the 11 principal components included in the adjusted analysis

| Eigenvalue Principal Component 1 (PC1) | 3.05 |
| --- | --- |
| Proportion of variance explained by PC1 | 0.27 |
| Scale reliability coefficient (Cronbach’s Alpha) | 0.71 |
| Kaiser-Meyer-Olkin sampling adecuacy-test | 0.72 |
