# Supplementary material for: Predicting adherence to postdischarge malaria chemoprevention in Malawian pre-school children: A prognostic multivariable analysis
Source: PLOS Glob Public Health. 2023 Apr 17;3(4):e0001779. doi: 10.1371/journal.pgph.0001779 (PMC10109490; doi:10.1371/journal.pgph.0001779)
Supplement: S2 Fig — PCA: Principal Component Analysis. (DOCX) [file pgph.0001779.s004.docx]

**Kühl et al: Predicting adherence to**

**postdischarge antimalarials in Malawian pre-school children**

**Supplementary Material, S2 Fig**

**S2 Fig:** Households’ relative socio-economic status based on adjusted PCA-analysis, separated into quintiles

Legend:

PCA: Principal Component Analysis
