# Supplementary material for: Predicting adherence to postdischarge malaria chemoprevention in Malawian pre-school children: A prognostic multivariable analysis
Source: PLOS Glob Public Health. 2023 Apr 17;3(4):e0001779. doi: 10.1371/journal.pgph.0001779 (PMC10109490; doi:10.1371/journal.pgph.0001779)
Supplement: Attachment — Submitted filename: Response to Reviewers.docx [file pgph.0001779.s005.docx]

PGPH-D-22-01351
Predicting caregivers’ adherence to postdischarge malaria chemoprevention in Malawian pre-school children: a prognostic multivariable analysis
PLOS Global Public Health

Dear Editor, att. Ms. Ashton

Thank you for allowing us to resubmit our manuscript ‘Predicting caregivers’ adherence to postdischarge malaria chemoprevention in Malawian pre-school children: a prognostic multivariable analysis’. On behalf of all authors, I thank you and the reviewers for the thorough reading and responses.

We appreciate both the editor’s and reviewers’ constructive comments. We agree with most of them, which led us to conduct a full reanalysis. It did not substantially change the direction of our findings. However, moving from odds ratio- to risk ratio-estimates provided more conservative and precise effect estimates for our outcome, as one of the reviewers had pointed out.

We resubmit now a revised version of the manuscript with most of the suggestions adopted, most notably a revised statistical analysis. Below we provide a point-by-point response to each reviewer. We believe that the review has notably improved the manuscript and we look forward to hearing from you.

Yours sincerely,

Melf Kühl
Corresponding author

**Feedback to the reviewers (our responses are written in blue):**

**Reviewer #1:**

Introduction
The authors indicated the need for this study and included very relevant and recent publication in this area. PDMC has been shown to reduce post-discharge mortality and hospital readmission among children with maaria/anaemia and therefore improving its adherence should be encouraged by all means.

Thank you for this comment. The content remains the same with improvements made based on suggestions from co-authors and the other reviewers.

Methods
-The authors need to add more details about the current study. I think they should make clear this was a secondary analysis of previous cluster randomized trial (page 4 lines 9/17).

We agree and have now clarified this. The first sentence of the paragraph now reads:

*“This study is a secondary analysis of data that was collected in the PDMC trial conducted in Malawi, described elsewhere in detail.”* (p. 4, l. 9; the following sentences specify it as cluster-randomized controlled trial).

-The sample size was powered for original trial end point. Do you think it was adequate to answer the new question on adherence? Maybe a post-hoc power could have helped detect if the study was adequately powered.

The trial’s primary outcome was ‘adherence’, and the sample size was determined for that analysis. It considered estimates of annual incidence of severe malaria and intra-cluster correlation calculations at an estimated 80% power to detect specified adherence increases per arm (Gondwe et al, 2018, Ref. 8 in the manuscript). For this predictor study, we maintained this outcome and pooled data from the trial’s factor-designed five arms to obtain the two main arms from the trial.

Our outcome ‘adherence’ is a proportion (in our case 70% of 357 caregivers being adherent). Each predictor thus could hypothetically be post-hoc tested. This is not meaningful, in our view, but we acknowledge the risk of a type 2 error for some of the relationships, and we mentioned this in the discussion (p. 14, l. 24). Our crude estimates have a relatively acceptable precision, which indicates that we can be relatively confident about the findings. Nonetheless, we agree with you - in view of our results - and suggest in the discussion that future studies consider larger samples or pooled analyses for a more thorough understanding of the complexity of adherence to prevention in children (p. 15, l. 7.)

-The original design was a cluster randomized trial, why was the initial design ignored in the prognostic model. I think not accounting for the clustering in the original trial was grave mistake in this modelling.

We had done all initial analyses adjusted and non-adjusted for clustering, in parallel. We obtained the same results and thus deliberately excluded clustering in the initial manuscript due to these results and the non-insignificant ICC that was established in the trial. However, we agree it is more robust to include this design-based factor in all our analysis. We now report only clustering-adjusted analysis and results:

(Analysis:) *We used a generalized linear model (GLM) for the Poisson family with a log link and robust variance estimation adjusting for clustering and study arm allocation [19]. The statistical significance of categorical variables was tested per subgroup category and for the entire variable using Wald testing. The Intra-Cluster Correlation coefficient (ICC) in the trial analysis was found to be insignificantly small (0.000008) [8]. This also applies to this secondary analysis, where 357 caregiver-child pairs came from 301 clusters. (p. 7, l. 24)*

-Did the authors collect any new data for this study or all data were collected during the original trial (Page 6 lines 3/12). If data on adherence was collected after study completion, it could be subject to reporting bias. Some caregivers could have lost the blister packs.

‘Adherence’ was the main outcome, and all adherence data was collected during the trial in unannounced visits in the communities within days of scheduled course completion. All predictor data were likewise exclusively collected during the trial, however, exclusively at baseline, at the hospital, upon a caregiver/child’s enrollment in the trial. We clarified this now this in the text, replacing ‘study’ and ‘research’ with ‘trial’ throughout the Data Collection-section, more clearly distinguishing between the predictor study and the original trial. (p. 5, l. 17)

-The authors have listed the predictors explored in this study and report the predictors were based on UNICEF Extended Model of Care’s three categories. The model creates a hierarchy of variables from the enabling predictors to underlying and lastly the immediate predictors. The implication of this model is that there could be many interactions i.e some enabling predictors could directly impact adherence or indirectly through underlying or immediate predictors. The same would be true for underlying predictors. Using a `flat’ model like logistic regression may not adequate test these interactions. Structural equation modelling would have adequately addressed the interactions and different paths.

The data collection and our categorizing of variables adopted the UNICEF framework; however, rather as ordering principle, placing the child at the center, than as hierarchical framework. We made this more specific now, eliminating the hierarchical labels:

*“Potential predictors were reported along the UNICEF Extended Model of Care’s three categories: predictors focused on the child, predictors related to the caregiver and their behavior, and predictors pertaining to their household’s resources* [17, 18].” (p.4, l. 4)

We agree we do not use the framework to its full intended purpose by ignoring the hierarchy between the categories. Using this simpler model and now reporting relative risks, however, GLM analysis serves our objectives well, we believe, in spite of remaining a ‘flat’ approach. Motivated by your suggestion, we now report more clearly how we checked for collinearity and interaction. This reads as follows, now, in the methods:

*“We tested for interaction with age and sex of both child and caregiver in the initial analysis. We also tested the crude and adjusted analyses for each treatment arm separately in view of the strong treatment effect. All variables included in the final model were tested for multicollinearity.”* (p. 8, l. 9)

-Which variables and what method was used to create the household wealth index?

We amended the manuscript with supplementary materials now, including (a) a summary of the methods we followed to develop and adjust a socio-economic index for our sample population, (b) a table of the variables considered, as well as (c) a graph of the principal components’ eigenvalues, and (d) a graph showing the distribution of households in quintiles across the index from the adjusted analysis (see separately attached ‘Supplementary materials’). In the manuscript itself we summarize the SES development as follows:

*“Predictive factors related to household resources included a household’s socio-economic status (SES, in quintiles) based on an index of various assets, including household items, available resources, livestock possession, dwelling size, building materials, and sanitation facilities. We used principal component analysis to create this wealth index from these variables and multicollinearity analysis to adjust it further (Text and Table S1; Figures S1, S2).”* (p. 6, l. 15)

-Another predictor that probably would impact adherence is access to health care given one arm was receiving their drugs at the clinic. The authors collected data on travel time to the study hospital but this predictor was not included in the model.

Correct, we had removed it from the list but failed to remove it from the manuscript as ‘distance’ turned out to provide more reliable data. This has now been corrected.

-Was data on maternal depression available?

No, this data was not collected, and we agree post-natal depression may well be a predictor for (poor) adherence. Assuming a prevalence of 30% in Malawi at 9 months after birth (Stewart et al, 2008) and considering the wide age distribution of included children, up to 59 months at enrollment, there was likely only a small number of mothers experiencing PND at the time of trial enrollment: 27 of 357 children were younger than 10 months at enrollment. An inquiry of all mothers on their recollecting experiencing PND in the past could have been done, however, with some bias attached. PND is under-researched, and we regret that a more holistic approach implementing mental health in the data collection was not done.

Results
- I think it would be challenging to interpret the effect of household wealth index on adherence since the authors have not reported the asserts included in the score and the method of computing the score.

We agree, please see answer above describing the newly written section in the manuscript and supplementary materials on the index.

-Approximately 6% caregivers were not biological caregivers, who were they? Would it make sense to run sensitivity analysis with only biological caregivers?

We excluded all caregivers that were not the “main” caregiver due to their inability to provide reliable information on the household in question (a total of 8). 94% of the 357 included caregivers were mothers, all remaining caregivers were also family members, thus arguably biological caregivers. We specified the relationship of this group now as follows:

*“Most caregivers were mothers (94%), and the other caregivers were other family members. Their mean age was 29 years.”* (p. 9, l. 10)

-I suspect the model could not work because there were many highly correlated variables for example were all the child anthropometry like HAZ, WAZ, WHZ, stunting, wasting, underweight etc included in the model simultaneous?

We only included variables in the final model that showed a significant association (p=<0.05) in the initial analysis. None of the now six anthropometric variables (by cut-off or continuous) were thus included in the adjusted model. Collinearity was considered at the multivariable analysis-level.

-What was the discriminatory value of the multivariable model? How did the model perform? Did you calculate a measure like AUCs?

For the revised analysis, we calculated the more k-fold cross-validated area under the receiver operating characteristic (ROC)-curve:

(Methods:) “Model performance was assessed by calculating the k-fold cross-validated area under the receiver operating characteristic (ROC)-curve [22].” (p. 8, l. 12)

(Results:) “The model’s performance, adjusted for k-fold cross-validation was acceptable, with the mean area under the ROC-curve estimated to be 0.63.” (p. 12, l. 11)

-Looking at the model and predictors included in the model, is it correct to say thi is a caregiver predictors model? Both child and care giver predictors are included.

We maintain that this is a caregiver model because the outcome ‘adherence’ only describes adherence behavior of the caregivers; even though the trial enrolled caregiver-child pairs. Whether a child at this age takes a tablet is assumed to be initiated and/or under full control of their caregiver. Refusal or vomiting could, perhaps, be considered a crude form of children’s poor adherence; however, for the outcome “adherence” as we defined it, this is not of concern. Anytime a child vomited as a side-effect, the caregiver did in fact adhere to the regimen thus far and “used” a tablet. Even with a caregiver-focus, it is helpful to include “proxy”-predictors on the child to identify caregivers at relatively higher risk of non-adherence; for example, a child’s malnutrition or a previous malaria infection are plausible child-focused factors possibly associated with adherence. Arguably, these factors still say more about the caregiver than about their young child.

Discussion
-Although the authors report their findings are inconclusive, I think they did not put serious thought in modelling including predictors selection, selection of regression model etc.

We adopted your suggestions and replied above, point by point, to your thorough and detailed critique of our method.

-Logistic regression overestimates risk ratios when the prevalence >10%. Literature suggests alternative models to estimate relative risk rather than odds ratio. Here are some useful literature: <https://www.ncbi.nlm.nih.gov/pmc/articles/PMC3348192/>
<https://www.ncbi.nlm.nih.gov/pmc/articles/PMC2292207/>
<https://academic.oup.com/aje/article/157/10/940/290159>

This feedback is agreeable and made us change our analytical approach. We reviewed the referred and other literature. On this basis, we decided to reduce the risk of overestimation by odds ratios and changed our analysis to modified Poisson regression for clustered data. We therewith report more conservative relative risks (described in detail above). Thank you for this insightful comment!

-Alternative the first step in this case could have been to start with qualitative work about adherence before fixing prognostic model.

Yes. While we published some qualitative work on the acceptability of PDMC, alongside the trial, we agree that a better qualitative understanding of preventive therapy in households needs to be explored. We therefore conclude our Discussion, according to your suggestion:

*“Future implementation research may thus consider pooling or collecting a larger data sample to better address this. Additionally, qualitative inquiry on regimen experience and adherence motivators may further clarify some of our mixed results.”(p. 15, l. 5)*

*Thank you for your thorough review!*

**Reviewer #2:**

A good paper on the predictors of caregivers adherence with a good comprehension of the immediate, underlying and enabling predictor. However, the paper could be improved in the following ways;

1. In your methods you mentioned that the variables with p-values <0.2 would be included in the adjusted logistic regression, with the results indicated on Table 1. However, it is noted that some logistic results that met the criteria were not included in the adjusted results and some results which did not meet the criteria had adjusted logistic results. Either this was a minor error or oversight with the superscript labelling, or the criteria wasn’t followed strictly. Otherwise, please clarify why the criteria was not strictly followed.

This was a mistake in the superscript, thank you for noting it! We have reviewed our statistical analysis, as described in the responses to reviewer #1, above. We no longer eliminate potential predictors backwards but instead move directly from an initial analysis of any potential predictor, adjusted for treatment arm and clustering, to an adjusted multivariable model. Very inclusive predictor selection in the multivariable model do not alter the findings, however, introduce more noise in the model performance.

2. Most of the predictors were not significant as stand-alone variables. It would be better if an interaction term was explored between the categories of predictors. In a real-life situation, these predictors all interact to influence the behaviour of a caregiver. This could yield more significant results and it would be interesting to see how these predictors interact.

We now included the treatment arm-allocation and clustering into the crude analysis of all potential predictors. In addition, we tested interaction considering sex and age of both caregiver and child, and we conducted the crude and adjusted analyses for either treatment arm-cohort separately.

3. Please clarify the method used to calculate the socio-economic variable; was this done using factor analysis (principal component analysis)? What did you use to inform your asset index questionnaire? Was it the DHS? Or the country’s establish wealth index?

We have described the SES more precisely now in the manuscript (within the scope of the paper) and amended it with a detailed description of variables included, method applied, and distribution of households across wealth quintiles. Kindly see our detailed reply above to reviewer #1’s question regarding the wealth index – and the newly added supplementary material.

Also, you tried to differentiate between the access of water variable in the SES index and the community source of water, which is confusing as the community source of water and household access to that source of water, all make up the household’s access to water. This makes the community source of water variable redundant and hence insignificant as it is already accounted for in the SES index results. This also goes for the household’s access to electricity which is also part of the SES index. Suggest you either remove community source of water and household electricity, as separate variables, from the logistic regression or give a clearer explanation on how these variables differ from the SES index as predictors.

This has been corrected, and we are glad you pointed out two mistakes in the reporting. We had summarized the SES-components incorrectly in the text. There was in fact no duplication of variables in the analysis. We considered all (drinking) water-related variables as separate predictors, while keeping all sanitation-facilities within the index as household-related assets in the SES-index; this was not clear in the manuscript. Electricity was not included in the index, either, and this was wrongly reported, too. We clarified this distinction now (following the description of the assets included in the SES):

*“Household members owning their dwelling, being connected to the electricity grid, being able to rely on a regular income, and owning a bank account, were factors that we considered potentially important individual predictors for adherence. We therefore removed them from the index and tested them as separate predictors in this category. We also included community-related factors: the kind of drinking water source they used, its distance, and coverage of community-level malaria control efforts, particularly indoor residual spraying.” (p. 6, l. 20)*

*Thank you for your critical review!*

**Reviewer #3:**

My main suggestion is on the choice of outcome and specifically the decision to categorise and report adherence as a binary 'all or nothing' (p8, line 6-8) rather than considering one (or more) category of partial adherence. The mean number of tablets taken by the non-adherent group (x̄=4.7, SD=2, range=0 to 8) shows the vast majority of those did take at least some tablets, but that there was substantial variation, including those never took and those who took almost all.

While partial adherence is of course sub-optimal, I think doing this would make the analysis potentially more informative in identifying risk factors for poor adherence more clearly than it does currently, which the authors concede is of limited utility. It may (or may not!) be the case that by grouping many different sets of adherence patterns together some risk factors are being obscured.

Connected to this – I would strongly suggest that even if the authors chose not to take the analysis in this direction, that the patterns of adherence are reported in more detail. This could be very valuable from a programmatic perspective, even if no clear risk factors are identified. What exactly is going on in the ‘not full adherence’ group – is it that one dose is being taken in full, the second in part, then the third not at all; or is it one to two tablets of each dose? Or is it just a very mixed picture with no clear pattern. Even descriptive analysis would be valuable given PDMC is quite novel and help contextualise the analysis.

These are essential questions to understand PDMC adherence. We agree that the non-adherent group’s adhering to the wide range of zero to eight of nine tablets appears overly generalized. We had considered various outcome definitions, and for our main predictor analysis we still suggest keeping this perspective, mainly in view of the specific distribution of total tablets taken per child. This rationale is now illustrated in a newly included graph (Figure 2) and summarized in the manuscript, with reference to further discussion in the trial paper.

*“Out of the included 357 children/caregiver couples, 249 (70%) had full PDMC adherence, and 108 (30%) were categorized as “not adherent” (Figure 2). The non-adherent category mostly received either zero, three, or six out of the nine tablets, reflecting that missing doses often involved skipping entire monthly course(s) of three tablets rather than one or two days of a 3-day course [8].”(* p. 9, l. 17)

Nonetheless, we followed your suggestion to analyze this outcome in sub-groups, too. Due to the monthly courses of the PDMC-regimen, the next sensible grouping of non-adherence would be three categories of non-adherence vs. the full-adherent group (as used in [8].Gondwe et al, 2021, and [23].” Kühl at al, 2022). Following our main analysis, we thus conducted ordered logistic regression analyses for these sub-groups, as well as a comparison of the means of potential predictor across the four adherence categories.

(Methods:) *“Considering the wide distribution within the non-adherent group (zero to eight tablets taken) we created sub-categories, as defined in the previous PDMC trial and the cost-effectiveness analyses: no or low (zero to 2 tablets), medium (three to less than six tablets), and high (six to eight tablets) adherence (Figure 2) [8, 23]. We then conducted ordered logistic regression analysis for this categorical outcome, to test if this resulted in a different predictor selection.*” (p. 8, l. 17)

(Results:) *“The analysis with the non-adherent group separated into non-adherent sub-categories (high but not full, medium, and low or no adherence) did not yield significant predictors and we do not report this analysis.”* (p. 12, l. 13)

Accounting for the smaller sample size, we also inspected each potential predictor’s (p-values <0.2) mean prevalence across these groups. We neither found any important difference comparing the mean occurrence of potential predictors across these non-adherence groups, each compared to the fully adherent group. We did not report this.

The timing/order of non-adherence is discussed in the trial paper [8]. It is a factor that further diversifies the outcome within these already relatively small non-adherence groups. We have no evidence that timing of non-adherence affects the efficacy of PDMC. In our recent cost-effectiveness study, we have thus assumed a linear dose-effect relationship regardless of order [23].

Other comments:

Page 2, line 12 – why are these associations necessarily counter intuitive (and to who)? This is repeated (page 14). In the discussion section the authors even go on to speculate that those with lower levels of literacy may receive better counselling from healthcare workers and cite some evidence to support this. I think it would be more appropriate to describe this without value judgement (e.g., just the direction of association, is it positive or negative).

We removed our judgments as you suggested, here and below, from the text and use “mixed” rather than “counter intuitive” results. (p. 2, l. 11; p. 12, l. 20; p.13, l. 23, p. 15, l. 8, 15)

However, in the discussion, we think it is important to consider possible explanations for results and to embed our interpretation in the literature. In this case, we refer only to better-paid trial-personnel under controlled trial conditions giving greater attention to less educated participants, as they are required to by trial protocol, for example in the information and consent process. We do not venture to speculate that health care workers do the same in their public healthcare routines in Malawi; rather, we imply a possibly poor external validity for the direction of this predictor.

Page 3, line 1 – specifically, WHO recommended PDMC.

We agree, and changed it to “*World Health Organization (WHO) recommended PDMC*”…

Page 5, line 3 –should this read “their” as later on (page 9, line 10) it is specified that not all caregivers were mothers.

We changed it to “*their”*, thank you.

Page 7, line 18 – please clarify how the “asset-based index” was calculated (PCA?) and what specific covariates were included (“several household features” is not clear).

We have described the SES more precisely now in the manuscript (within the scope of the paper) and amended it with a detailed description of variables included, method applied, and distribution of households across wealth quintiles. Kindly see our detailed reply above to reviewer #1’s question regarding the wealth index – and the newly added supplementary material.

Page 8, line 11 – why would observed treatment (I presume this was a small number though) not be considered reliable way of measuring adherence?

We corrected this mistake by deleting the “or observed”. There was no observed treatment adherence in the trial, only unannounced blister pack collection in the community with pill count if not returned empty. (We initially used “observed" here in the sense of caregivers following (observing/respecting) their prescribed administration protocol.)

Page 13, line 3-5 – can you map these to standard categorisation of water source (e.g. how do (protected) dug well or spring fit into these)?

Our data distinguished seven categories (excluding ‘bottled water” which no household reported):

**main source of drinking water	Freq.	Percent	Cum.**

piped in dwelling	17	4.76	4.76

communal stand piped	38	10.64	15.41

hand pump/borehole	265	74.23	89.64

Private well	3	0.84	90.48

Public well	18	5.04	95.52

Spring	7	1.96	97.48

River/stream	9	2.52	100.00

**Total	357	100.00**

Matching these with UNICEF’s standardized five categories of drinking water sources (three improved subcategories, one unimproved, and surface water) is, unfortunately, problematic. We can assume piped water was always from improved sources. However, we have no reliable information whether the most accessed source (hand pumped or borehole water) was always structurally “improved”, and the same is true for wells. Spring and River/stream water can be equated to “surface water” (spring is debatable). With this uncertainty, we categorized the data in a way that improved water sources and surface water are reliably included in the analysis as categories 1 and 3, whereas the category in between likely includes both improved and non-improved sources. This is suboptimal but pragmatically useful for predictor analysis.

**Source of drinking water	Freq.	Percent	Cum.**

1 piped water	55	15.41	15.41

2 pumped groundwater	286	80.11	95.52

3 open water	16	4.48	100.00

**Total	357	100.00**

Referring to your example: a dug well could be ‘improved’ or ‘unimproved’ based on the lining and water outlet. In our categorization this would be category 2: ‘pumped groundwater’. A spring, unless with improved outlet, is the non-improved transition from ground to surface water. It would thus be category 3 here (now renamed ‘surface water’).

We agree this is a pragmatic categorization in view of the data, but we think it may still be useful to keep it and test it because the categories are useful indicators for the public health management within a household’s community. Notably, we reviewed our category names following your comment and renamed them to offer a clearer reference to UNICEF categories (Table 1):

| *Source of drinking water used by the household* | *piped water* ***(improved)*** |
| --- | --- |
|  | *pumped ground water* ***(improved and non-improved)*** |
|  | *Surface water* ***(non-improved)*** |

Page 14, line 23-25 – would the authors consider reporting adherence reported by self report and/or direct observation as a supplementary table?

There was no direct observation. We had phrased this wrongly in the manuscript (using “observed”). This has been corrected and responded to above.

We prefer the pill-count as adherence-outcome measure. While it has methodological problems in a community-setting it is still considered the gold standard for designs like this trial. While we have data on self-reported adherence, it introduces a widely documented unnecessary bias of over-reporting adherence that we chose to avoid. This decision is also based on the distribution between the two outcome groups; we have the possibility to rely on the hard measure ‘pill count’ for a behavior outcome. If there were many fewer fully adhering caregivers, the softer measure ‘self-reporting’ might merit more weight in the analysis.

Page 14-17 – Would the authors consider citing evidence about adherence to treatment from outside malaria that could be relevant to this discussion more broadly? For example, I would be surprised if there was not evidence from community use of antibiotics etc (see Non-adherence to oral antibiotics for community paediatric pneumonia treatment in Malawi – A qualitative investigation).

Thank you, the study on antibiotics is indeed interesting and introduces one dimension we have not covered: community-based pressures to share medication. We are aware of this practice; however, it was not captured by our predictors or in our ‘hard’ outcome when collecting the empty blister packs.

In terms of comparability, the key feature that distinguishes PDMC is that it is given preventively; therefore, comparable regimens would need to be preventive in kind, and ideally of similar “inconvenience” to caregivers and children, administered non-observed, preferably at comparably infrequent intervals. All of these drive behavior (mainly: not forgetting). Treating a sick – and then improving child – over a few consecutive days for pneumonia (or malaria) is hard to compare. This sickness and the immediate power to alleviate it create a relatively stronger reminder and incentive, respectively, to adhere to it – over a few consecutive days. We therefore rejected comparing DP with curative regimens – even for malaria – when aiming to understand drivers of adherence. We summarize this argument on p. 14, from l. 6 onwards.

Page 15, line 13 – I am not sure this sentence is necessary, as the authors go on to give a reasonable explanation based on the literature.

Page 15, line 21-22 – I am not sure the word “mastered” is appropriate in this context, perhaps “widely spoken”. (p. 13, l. 20)

Page 16, line 3 – again, I am not sure the word “inexplicable” is the best choice here, “mixed” is sufficient. (See above)

We adopted your suggestions for these three points.

Thank you for this thorough review!

***References (not included in the manuscript):***

1. **Stewart RC**, Umar E, Kauye F, Bunn J, Vokhiwa M, Fitzgerald M, Tomenson B, et al. Maternal common mental disorder and infant growth--a cross-sectional study from Malawi. Matern Child Nutr. 2008 Jul;4(3):209-19. doi: 10.1111/j.1740-8709.2008.00147.x.
