# Supplementary material for: Predicting adherence to postdischarge malaria chemoprevention in Malawian pre-school children: A prognostic multivariable analysis
Source: PLOS Glob Public Health. 2023 Apr 17;3(4):e0001779. doi: 10.1371/journal.pgph.0001779 (PMC10109490; doi:10.1371/journal.pgph.0001779)
Supplement: Attachment — Submitted filename: Response to Reviewers.docx [file pgph.0001779.s006.docx]

PGPH-D-22-01351
Predicting adherence to postdischarge malaria chemoprevention in Malawian pre-school children: a prognostic multivariable analysis
PLOS Global Public Health

Dear Editor, att. Ms. Ashton

Thank you for asking us to resubmit our manuscript ‘Predicting adherence to postdischarge malaria chemoprevention in Malawian pre-school children: a prognostic multivariable analysis’ with minor revisions.

We appreciate the reviewer’s thorough review and constructive suggestions. We agree with all of them and resubmit a revised version of the manuscript with all his suggestions adopted. We also changed the title, as suggested, in the manuscript and throughout the submission documents.

Below we provide a brief point-by-point response to the reviewer. We hope with the revised title and minor changes in the text we satisfy your journals publication criteria.

Yours sincerely,

Melf Kühl
Corresponding author

**Feedback to the reviewer (our responses are written in blue):**

**Reviewer #1, Dr Moses Ngari:**

Thank for addressing the comments, the manuscript reads much better now. However, I have one major and minor comments.
***Major comment***
I am still struggling with the title of the study. I would remove the word 'caregiver' from the title so that it reads `Predicting adherence to PDMC ....'. It is because we are talking about PDMC on children and not caregivers.

Thank you for this suggestion. We agree that, especially in the title, the mention of caregivers may be misleading. We follow your suggestion to shorten the full title, as follows:

*Predicting adherence to postdischarge malaria chemoprevention in Malawian pre-school children: a prognostic multivariable analysis* (p. 1)

We applied the same to the short title:

*Predicting adherence to postdischarge antimalarials in Malawian pre-school children* (p. 1)

***Minor comments***
a) Page 11, line 7 on the results, the author report the measure of effect PR rather than RR (PR, 95% 0.65, 0.55 to 0.76).
b) Page 12, line 7, are the measures of effect OR or RR?

Thank you for highlighting these mistakes in the results. Clearly, you are right, and we corrected both mistakes to now read ‘RR’:

(a) … *compared to community-based PDMC (RR, 95% CI: 0.64, 0.55 to 0.76).* (p. 11, l. 6)

(b) … *with higher non-adherence (RR, 95% CI: 0.78, 0.64 to 0.95; and 0.79, 0.67 to 0.92, respectively).* (p.12, l.7-8)

Alongside these two mistakes, which were remainders from an earlier analysis, we also corrected the wrong values in (b) that we had also failed to update in that paragraph. They now match the final analysis, as reported in table 1.

c) Can you add 95%CI to the AUC? You could use bootstrapped AUC (say with 1000 replications for internal validation).

Thank you, this is important. We follow your suggestion and have now included statistical inference in the text of both the methods and results-section:

Methods:

*(…) Model performance was assessed by calculating the k-fold cross-validated area under the receiver operating characteristic (ROC)-curve with statistical inference obtained by bootstrapping [22].* (p. 8, 12-14)

Results:

*(…) The model’s performance, adjusted for k-fold cross-validation, was acceptable, with the mean area under the ROC-curve estimated to be 0.65 (95%CI: 0.57 to 0.71).* (p. 12, l. 12)

Thank you for this thorough review!
